# Building a Full-Atom Model of L,Dtranspeptidase 2 from Mycobacterium tuberculosis for Screening New Inhibitors

**Published:** 2017

**Authors:** S.M. Baldin, N.M. Misiura, V.K. Švedas

**Affiliations:** Belozersky Institute of Physicochemical Biology, Lomonosov Moscow State University, Leninskie gory 1, bldg. 40, Moscow, 119991, Russia; Faculty of Chemistry, Lomonosov Moscow State University, Leninskie gory 1, bldg. 3, Moscow, 119991 , Russia; Faculty of Bioengineering and Bioinformatics, Lomonosov Moscow State University, Leninskie gory 1 , bldg. 73, Moscow, 119991, Russia

**Keywords:** L,D-transpeptidase, Mycobacterium tuberculosis, catalytic mechanism, molecular docking, molecular dynamics simulations, inhibitors

## Abstract

L,D-transpeptidase 2 from *Mycobacterium tuberculosis *plays a key role in the formation of the cell wall of a pathogen and catalyzes the cross-linking of growing peptidoglycan chains by non-classical 3-3 bonds, which causes resistance to a broad spectrum of penicillins. Molecular modeling of enzyme interactions with the N- and C-terminal tetrapeptide fragments of growing peptidoglycan chains has been performed for the first time and has allowed us to highlight the peculiarities of their binding at the formation of 3-3 cross-linkages, as well as to build a full-atom model of L,D-transpeptidase 2 for the screening and optimizing of inhibitors’ structures.

## INTRODUCTION

The danger posed by tuberculosis continues to grow with the appearance of new multidrug-resistant strains of *M. tuberculosis*. According to the WHO report of 2015, approximately 10.4 million people contracted tuberculosis and 1.8 million people died from it [1]. There is an obvious need for new anti-TB drugs, as well as medical treatment technologies, not to mention the design of more effective antibiotics to suppress the infection. That is why new, heretofore unknown, molecular targets that are associated with the functioning and structural organization of the causative agents of tuberculosis are of particular interest.

**Fig. 1:**
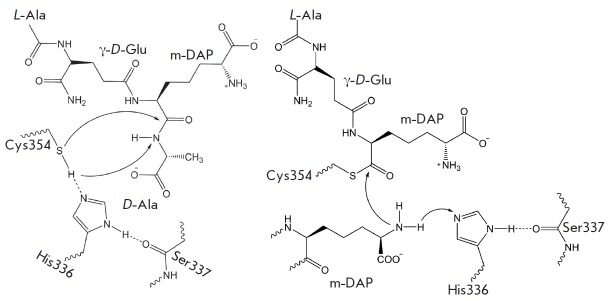
Catalytic mechanism of the LdtMt2 is shown with the tripeptide analog of the natural substrate. Formation of the acyl enzyme is presented in the left part, the subsequent acyl transfer to the nucleophile and the formation of 3-3 cross-linkage of peptidoglycan is presented in the right part of the figure

One of the essential distinctive features of *Mycobacterium tuberculosis *is the structure of its cell wall. In most bacteria, a cell wall contains classical 4-3 cross-linkages of peptidoglycan chains (bonds between the meso-diaminopimelic acid (m-DAP) residue and D-Ala). At the same time, the cell wall of *M. tuberculosis *for the most part originates from the formation of non-classical 3-3 cross-linkages (up to approximately 80% of all bonds between m-DAP residues of different peptidoglycan chains in the stationary phase). Once this was discovered, it became clear why β-lactam antibiotics capable of inactivating penicillin-binding enzymes such as D,D-transpeptidases that catalyze the formation of classical 4-3 cross-linkages [2] are ineffective in the treatment of tuberculosis. It was recently established that the formation of non-classical 3-3 cross-linkages is catalyzed by the earlier unknown enzymes L,D-transpeptidases (*Fig. 1*).

The genome of *M. tuberculosis *encodes five proteins which contain L,D-transpeptidase domains (sites Rv0116c, Rv0192, Rv0483, Rv1433 and Rv2518c) [5]. Rv2518c, which codes LdtMt2, is the most abundantly expressed gene. Loss of this gene leads to changes in colony morphology, suppresses the growth of bacteria, and increases sensitivity to classical antibiotics (amoxicillin used in combination with clavulanic acid) [3].

LdtMt2 is a lipoprotein that consists of 408 amino acid residues; its N-terminal region is located in a lipid bilayer. The polypeptide chain contains a short region exposed inside the cell, a transmembrane region, and a region exposed outside the membrane. The outside region can be divided into 3 domains: domains A and B, which are non-catalytic IG-like domains (comprising residues 55-146 and 149-250, respectively), and the C-terminal catalytic domain C (residues from 251 to 408) [6]. Residues Cys354, His336, and Ser337 play a key role in the catalysis and constitute a catalytic triad [6]. The active site of LdtMt2 is isolated from the solvent and hidden under the so-called active site lid (residues Tyr298-Trp324) that forms 3 tunnels, A, B & C, the last two being involved in a process of substrate delivery to the active site [7]. This element of secondary structure represents an antiparallel β-sheet with a disordered loop. Access of the substrate and solvent to the active site is limited by bulky Tyr308 and Tyr318 residues, which are part of the lid, as well as the Tyr330, Phe334, and Trp340 residues that are located at the entrance to the active site.

**Fig. 2:**
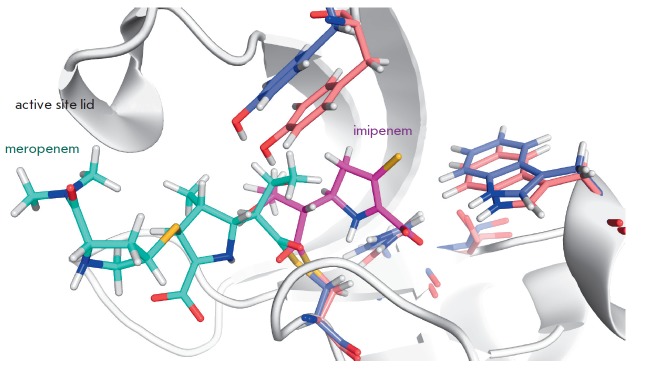
Structural alignment of the enzyme-inhibitor complexes LdtMt2-meropenem (4GSU) and LdtMt1-imipenem (4JMX) demonstrates that residues of meropenem (blue) and imipenem (pink) are located in different tunnels

The full-atom structure of LdtMt2 remains unknown. However, the structures of the domains A&B and B&C (PDB, 4HU2 & 4HUC, respectively) are available [6]. Furthermore, the structures of the catalytic domain with the dipeptide fragment N-γ- D-glutamyl-m-DAP of peptidoglycan (PDB 3TUR) [5], as well as the covalent complexes of LdtMt2 with meropenem and LdtMt1 with imipenem, have been reported [7, 8]. Confusingly, there is a discrepancy between the presented structures: molecules of the same class of inhibitors (meropenem and imipenem) are located in different tunnels despite the high homology of the catalytic L,D-transpeptidase domains in LdtMt1 and LdtMt2 (*Fig. 2*).

Important information concerning the catalytic mechanism of LdtMt2 was obtained during QM/ MM-modeling of the enzymatic reaction with the tripeptide fragment N-γ-D-Glu-m-DAP-D-Ala of peptidoglycan [9]. The authors identified the energy profiles for two reaction stages: the formation of the acyl enzyme and the consequent acyl (L-center of the first m-DAP residue) transfer to the nucleophile (D-center of the second m-DAP residue) that leads to the cross-linking of peptidoglycan chains (*Fig. 1*). Molecular modeling techniques may help identify the structural peculiarities in the active site organization of L,D-transpeptidases, their interaction with substrates, and facilitate the search for inhibitors. In order to achieve this, it is necessary to be in possession of adequate molecular models of the enzyme that can be used to screen libraries of potential inhibitors. The goal of this work was to perform a molecular modeling of enzyme binding with the tetrapeptide fragment of peptidoglycan, as well as with β-lactam compounds, and to build a full-atom model of LdtMt2 for the screening and optimization of inhibitors’ structures.

## EXPERIMENTAL SECTION

**Software**

Calculation of the ionization states of amino acid residues was performed by PROPKA 3.0 [10]. The starting structure preparation and trajectory analysis were performed using the AmberTools 16 program package, and energy minimization and molecular dynamics were calculated in Amber 14 [11]. Molecular docking was performed with AutoDock 4 [12]. The structures of the inhibitors and substrate fragment were built using ACD/ChemSketch (*http://www.acdlabs.com*). Geometry optimization and calculation of partial atomic charges were carried out with PC GAMESS/Firefly 8.1 [13] and the resp module of AmberTools 16, respectively. Visualization and structure editing of enzyme-substrate complexes were performed by VMD 1.9.2 [14] and PyMol 1.8 [16]. Computation of molecular dynamics trajectories was performed using the MSU supercomputer “Lomonosov” [16].

**Structure preparation**

Two crystallographic structures were selected as starting models of the enzyme and enzyme-substrate complex: 4HU2 [6], which contains coordinates of the non-catalytic domains A and B; 3TUR [5], which contains coordinates of the catalytic domain C, as well as the non-catalytic domain B and the dipeptide fragment of peptidoglycan of the *M. tuberculosis *cell wall (γ-D-Glu-m-DAP). A full-atom model of the LdtMt2-substrate complex was built according to the following procedure: two structures were aligned via domain B and the chains were connected to form a three-domain structure of LdtMt2, then the missing amino acid residues D-Ala and L-Ala in a peptidoglycan fragment were added to the substrate’s structure, and the N-acetyl group was attached to the L-Ala residue in order to neutralize the charge of the N-terminal amino group. Partial atom charges were determined as follows: the initial geometry of each amino acid residue was optimized at the MP2/6-31G* level of theory, then the electrostatic potential was calculated according to HF/6-31G*. The resulting structure of the enzyme-substrate complex was placed in a TIP3P water cell with a minimum distance of 10 Å from the edge of the cell. Calculation of molecular dynamics trajectories was performed with AMBER ff14SB [17]. The constants of the force field for the substrate’s bonds and angles were obtained from ff14SB and other missing parameters, from GAFF.

**Energy minimization and molecular dynamics**

Models of the enzyme and enzyme-substrate complex were equilibrated, and molecular dynamics’ trajectories were calculated according to the following protocol: first, two steps of the energy minimization of the system were performed. The energy of the solvent residues was minimized by the steepest descent algorithm (2,000 steps), followed by a conjugate gradient algorithm (2,000 steps). Afterwards, the system was heated up at a constant volume from 0 to 310K over 50 ps and then equilibrated over 1,000 ps under constant pressure. The integration step was 0.002 ps. All simulations were performed using periodic boundaries and the Particle Mesh Ewald method (PME) to account for long-range Coulomb interactions. The radius of the cut-off disconnected Coulomb interactions was 8 Å.

**Molecular docking**

The LdtMt2 model to perform the molecular docking of different β-lactam compounds was prepared as follows: water molecules, sodium ions, and the substrate (fragment of peptidoglycan) were removed from the solvated structure of the enzyme-substrate complex taken from the molecular dynamics trajectories after energy minimization. Then, a map of potential interactions was calculated around the active site and the β-lactam inhibitors were docked using the Lamarckian genetic algorithm. A series of 50 to 100 independent docking runs was performed for each compound. The resulting enzyme-inhibitor complexes were clustered, and the peculiarities of the structural organization of the enzyme-inhibitor complexes in each cluster were analyzed. We considered the following indicators as a criterion of productive binding of an inhibitor in the enzyme active site that leads to a reactive enzyme-inhibitor complex and then the formation of a stable acyl enzyme:

1. The formation of hydrogen bonds between the inhibitor and the enzyme in the oxyanion hole (formation of hydrogen bonds between the N-atoms of the main chain formed by the residues Gly353 and Cys354 with the carbonyl oxygen of the β-lactam ring);

2. The distance between the attacking sulfur atom Sγ of catalytic cysteine Cys354 and the C atom of the carbonyl group of the β-lactam ring does not exceed 5 Å.

## RESULTS AND DISCUSSION

**Molecular modeling of peptidoglycan binding in alternative site of LdtMt2**

**Fig. 3:**
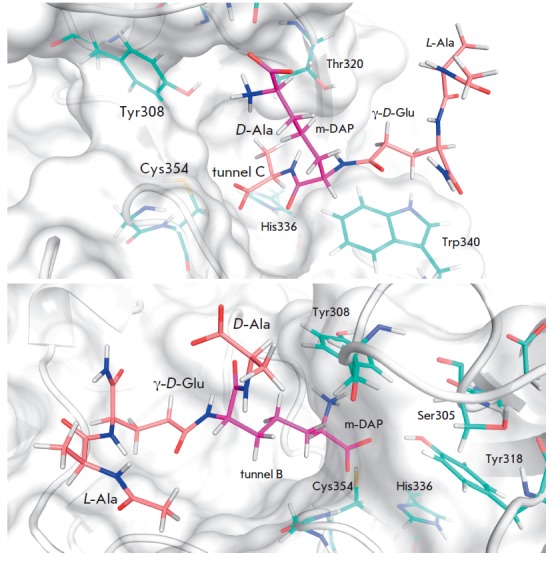
Interaction of the tetrapeptide fragment of peptidoglycan with the LdtMt2 active site residues at substrate binding in tunnel C (upper panel) and the tunnel B (lower panel)

The difficulty in determining substrate localization in the L,D-transpeptidase active site is further compounded by the fact that binding of two molecules of the same compound should be considered. One molecule should bind as the acyl donor, which further leads to the formation of an acyl enzyme intermediate, whereas the other one should bind as a nucleophile, which leads to the formation of the acyl enzyme-nucleophile complex, followed by the acyl group trans fer to the nucleophile and 3-3 cross-linking of peptidoglycan of the cell wall. The discrepancy in earlier published results concerning the localization of substrate-like inhibitors in the active site of LdtMt2 and LdtMt1 can result from insufficient attention paid to the opportunities of two different ways of binding of the natural substrate. In order to search for an optimal structure of the covalent LdtMt2 inhibitors (such, for example, as β-lactam compounds carbapenems), it is necessary to proceed with an adequate structure of the enzyme-substrate complex, where the substrate molecule occupies the position of the acyl donor capable of forming the acyl enzyme. Models of alternative binding of the tetrapeptide fragment (N-Ac–L-Ala–γ-D-Glu– m-DAP–D-Ala) of the natural substrate in the LdtMt2 active site were built on the basis of the 3TUR structure, and molecular dynamics was applied to discriminate between two different ways of substrate binding in the active site. In the first model, the tetrapeptide N-Ac–L-Ala–γ-D-Glu–m-DAP–D-Ala was placed in tunnel C, whereas in the alternative model the tetrapeptide was located in tunnel B (*Fig. 3*).

At binding of the peptidoglycan fragment in tunnel B, the substrate hydrogen bonds are formed with the residues His352, Ser331, Tyr308 and Tyr318. In the model of the enzyme-substrate complex with the substrate positioned in tunnel C, labile hydrogen bonds form with the residues Asn356, Trp340, His352, and Tyr318, similarly to the binding of the shorter tripeptide analog of the natural peptidoglycan [9]. Our simulations show that the L-Ala residue in a tetrapeptide fragment of peptidoglycan does not play a key role in the substrate binding in both (B and C) tunnels, and it is exposed to the solvent most of the time during MD trajectories. However, it is capable of forming short-lived hydrogen bonds with the N- and O-atoms of a backbone formed by the residues Arg319 and Thr320. This fact allows us to suggest that the residues of N-acetylglucosamine and N-acetylmuramic acid next to L-Ala in a peptidoglycan structure do not participate (or play a minor role) in the recognition of the acyl donor by the enzyme.

**Fig. 4:**
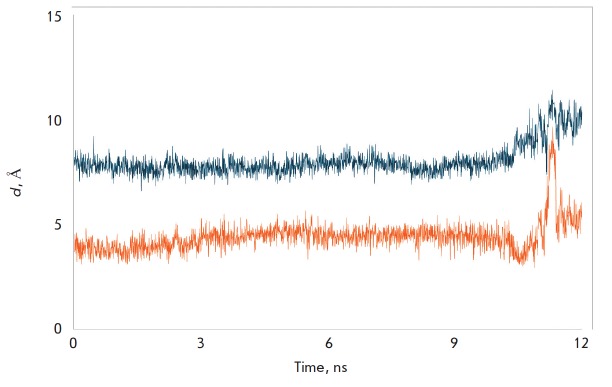
MD simulation of the tetrapeptide binding in the tunnel C of LdtMt2. Characteristic distances between the Sγ atom of Cys354 and the C atom of the carbonyl group of the peptide m-DAP-D-Ala (orange line), and the N-atom of the D-center of m-DAP (blue line)

**Fig. 5:**
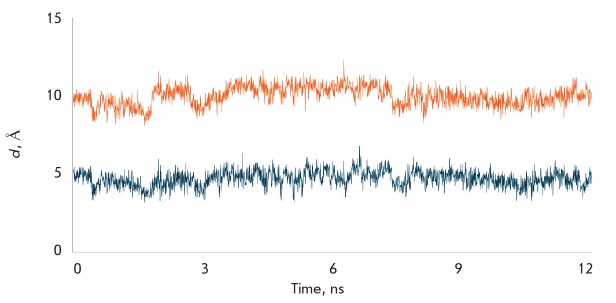
MD simulation of the tetrapeptide binding in the tunnel B of LdtMt2. Characteristic distances between the Sγ atom of Cys354 and the C atom of the carbonyl group of the peptide m-DAP-D-Ala (orange line), and the N-atom of the D-center of m-DAP (blue line)

At binding of the substrate in tunnel C, a reactive enzyme-substrate complex is formed: the Sγ-atom of the catalytic Cys354 residue is at a distance (3.5–5.7 Å) favorable for a nucleophilic attack of the C atom of the carbonyl group of the peptide bond m-DAP-D-Ala during the whole MD trajectory (*Fig. 4*, orange line). It should be noted that in a model of the enzyme-substrate complex when the peptidoglycan fragment is located in tunnel B, the substrate is positioned differently and is incapable of forming an acyl enzyme. The distance between the Sγ-atom of the catalytic Cys354 residue and the C atom of the carbonyl group of the peptide bond m-DAP-D-Ala during the whole MD trajectory (*Fig. 5*, orange line) varies in the range 8.1– 12.3 Å, which excludes the possibility of this substrate molecule playing the role of acyl donor. At the same time, the orientation of the substrate molecule in tunnel B well corresponds to the role of the nucleophile at the formation of the 3-3 cross-linkage: the distance between the N-atom of the free amino group of m-DAP and the Sγ-atom of catalytic Cys354 is in the range 3.2–6.8 Å (*Fig. 5*, blue line). Simulations have shown that an alternative role for each substrate molecule is impossible: the tetrapeptide positioned in tunnel C cannot serve as the nucleophile (*Fig. 4*, blue line), and the tetrapeptide bound in tunnel B cannot play the role of an acyl donor (*Fig. 5*, orange line). Establishment of this fact is important both for understanding the full catalytic cycle of the enzyme, and for searching for its inhibitors. Thus, at a structural optimization of β-lactam inhibitors capable of inactivating the enzyme due to the formation of a stable acyl enzyme, it is necessary to consider the binding of the inhibitor in tunnel C: i.e., in the location where binds the substrate molecule that plays the role of an acyl donor at the 3-3 cross-linking of the peptidoglycan chains.

**Modeling of the interaction of LdtMt2 and β-lactam compounds**

In two reported structures of acyl enzymes (4JMX formed at the inactivation of LdtMt1 by imipenem and 4GSU formed at the inactivation of LdtMt2 by meropenem), the residues of the inhibitors are located in different tunnels of the active site. The meropenem residue in the LdtMt2 active site is positioned at the entrance to tunnel B and mostly exposed to a solvent, whereas the thiazoline ring of the imipenem residue in the active site of LdtMt1 is entirely immersed in tunnel C.

**Fig. 6:**
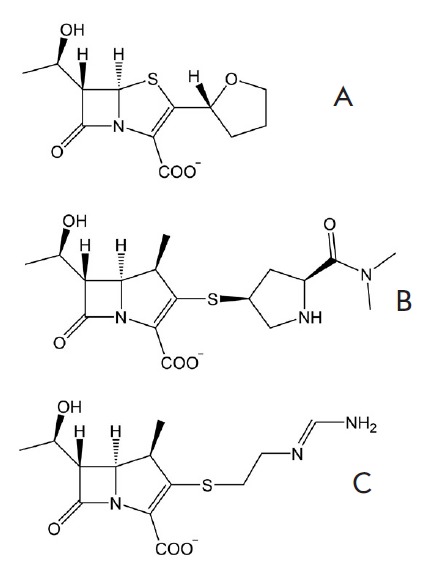
Structures of β-lactam antibiotics that were docked in the active site of LdtMt2. [A] - faropenem, [B] - meropenem, [C] - imipenem

In this case, it is important to determine the primary location of the β-lactam ring in the active site, which could correspond to the reactive enzyme-inhibitor complex capable of forming the acyl enzyme. Based on the criteria of theoretical chemistry, the nucleophilic attack on the C atom of the carbonyl group of the β-lactam ring by the Sγ atom of the catalytic Cys354 residue of LdtMt2 may materialize only if the distance between these atoms is in the range 3.5–4.0 Å and if the carbonyl group of the β-lactam ring is located in the oxyanion hole. Basing on our molecular modeling results of enzyme binding with the tetrapeptide fragment of natural peptidoglycan, we performed the molecular docking of three β-lactam inhibitors known from the literature that were shown to form reactive enzyme-inhibitor complexes with LdtMt2 (*Fig. 6*). Like in the modeling of enzyme-substrate interactions, we considered the binding of inhibitors in both tunnel B and tunnel C.

**Fig. 7:**
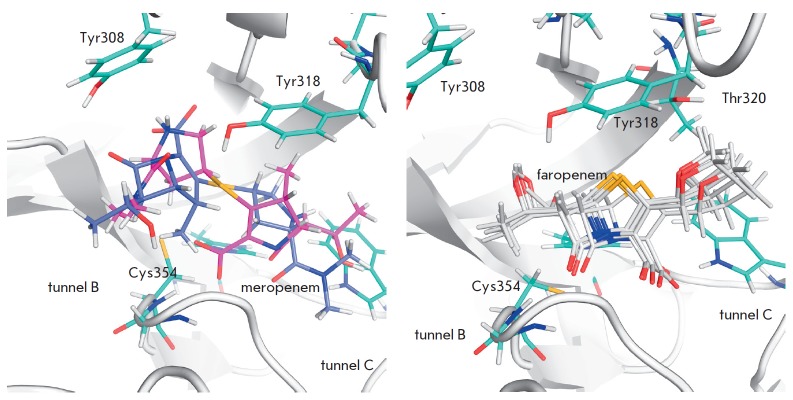
Localization of β-lactam antibiotics in the LdtMt2 active site at meropenem binding in the tunnel B (left panel) and faropenem binding in the tunnel C (right panel). In case of meropenem there is no accurate place for binding of the inhibitor and formation of the reactive enzyme-inhibitor complex, a distance between the Sγ-atom of the catalytic Cys354 and the C atom of the carbonyl group of β-lactam ring is not optimal for nucleophilic attack. At faropenem binding in the tunnel C (right panel) all docked structures form a single cluster that correspond to the reactive enzyme-inhibitor complex. The distance between the Sγ atom of the catalytic Cys354 and the C atom of the carbonyl group of β-lactam ring is optimal for nucleophilic attack and the carbonyl group of β-lactam ring can form hydrogen bonds with the oxyanion hole residues

At docking in tunnel B, we observed no formation of reactive enzyme complexes with any of the tested inhibitors: the distance between the Sγ atom of the catalytic Cys354 of LdtMt2 and the C atom of the carbonyl group of the β-lactam ring exceeded 5 Å. Binding in tunnel B also did not allow the carbonyl group of the β-lactam ring to form hydrogen bonds with the oxyanion hole residues (main chain N-atoms of Cys354 and Gly353). A typical example of the LdtMt2- meropenem complex is shown in *Fig. 7* (left). Thus, we concluded that an inhibitor cannot form a reactive complex with the enzyme at binding in tunnel B.

## CONCLUSIONS

The main goal of this work was to study binding of the tetrapeptide fragment of the natural substrate - cell wall peptidoglycan in the LdtMt2 active site and build a full-atom model of the enzyme-substrate complex which could allow one to search for new substrate-like irreversible inhibitors and to optimize their structure. The conducted molecular dynamics simulations have shown that binding of the N- and C-terminal fragments of the growing peptidoglycan chain in different tunnels is responsible for the different steps of the catalytic mechanism at the formation of non-classical 3-3 cross-linkages in peptidoglycan. In order to simulate LdtMt2 interaction with β-lactam inhibitors capable of inactivating the enzyme through the formation of stable acyl enzymes, it is necessary to consider the binding of potential inhibitors in tunnel C of the active site.

## Abbreviations

Ac: acetyl
WHO: World Health Organization
Ldt: L,D-transpeptidase
LdtMt1 and LdtMt2: L,D-transpeptidase 1 and 2 from Mycobacterium tuberculosis
m-DAP: meso-diaminopimelic acid
IG: immunoglobulin,
MD: molecular dynamics
PME: Particle Mesh Ewald

## References

[1] (2016). WHO. Global Tuberculosis Report; Geneva, 2016..

[2] Fisher J.F., Meroueh S.O., Mobashery S. (2005). Chem. Rev..

[3] Gupta R., Lavollay M., Mainardi J., Arthur M., Bishai W., Lamichhane G. (2010). Nat. Med..

[4] Jankute M., Cox J.A., Harrison J., Besra G.S. (2015). Ann. Rev. Microbiol..

[5] Erdemli S., Gupta R., Bishai W.R., Lamichhane G., Amzel M., Bianchet M. (2012). Structure..

[6] Böth D., Steiner E.M., Stadler D., Lindqvist Y., Schnell R., Schneider G. (2013). Acta Crystallogr. D Biol. Crystallogr..

[7] Kim H.S., Kim J., Im H.N., Yoon J.Y., An D.R., Yoon H.J., Kim J.Y., Min H.K., Kim S.J., Lee J.Y. (2013). Acta Crystallogr. D Biol. Crystallogr..

[8] Correale S., Ruggiero A., Capparelli R., Pedone E., Berisio R. (2013). Acta Crystallogr. D. Biol. Crystallogr..

[9] Silva J.R.A., Roitberg A.E., Alves C.N. (2014). J. Chem. Inf. Model..

[10] Li H., Robertson A.D., Jensen J.H. (2005). Proteins..

[11] Case D.A., Berryman J.T., Betz R.M., Cerutti D.S., Cheatham T.E., III I.O., Darden T.A., Duke R.E., Giese T.J., Gohlke H., Goetz A.W. (2015). AMBER 2015, University of California, San Francisco.

[12] Morris G.M., Huey R., Lindstrom W., Sanner M.F., Belew R.K., Goodsell D.S., Olson A.J. (2009). J. Comput. Chem..

[13] Granovsky A.A. Firefly version 8.. classic.chem. msu.su/gran/firefly/index.html.

[14] Humphrey W., Dalke A., Schulten K. (1996). J. Mol. Graphics..

[15] The PyMOL Molecular Graphics System. Version 1.8 Schrödinger, LLC..

[16] Voevodin V.V., Zhumatiy S., Sobolev S., Antonov A., Bryzgalov P., Nikitenko D., Stefanov K., Voevodin V. (2012). Open Systems J..

[17] Maier J.A., Martinez C., Kasavajhala K., Wickstrom L., Hauser K.E., Simmerling C. (2015). J. Chem. Theory Comput..

